# BPA Alters Estrogen Receptor Expression in the Heart After Viral Infection Activating Cardiac Mast Cells and T Cells Leading to Perimyocarditis and Fibrosis

**DOI:** 10.3389/fendo.2019.00598

**Published:** 2019-09-04

**Authors:** Katelyn Ann Bruno, Jessica Elizabeth Mathews, Alex Lingyun Yang, J. Augusto Frisancho, Ashley Jennie Scott, Henry David Greyner, Frank Anthony Molina, Merci Shekinah Greenaway, George Maxwell Cooper, Adriana Bucek, Andrea Carolina Morales-Lara, Anneliese Ruth Hill, Anna Alisa Mease, Damian Nicolas Di Florio, John Michael Sousou, Alexandria Christine Coronado, Allison Ray Stafford, DeLisa Fairweather

**Affiliations:** ^1^Department of Cardiovascular Medicine, Mayo Clinic, Jacksonville, FL, United States; ^2^Center for Clinical and Translational Science, Mayo Clinic, Jacksonville, FL, United States; ^3^Department of Immunology, Mayo Clinic, Jacksonville, FL, United States; ^4^Department of Environmental Health Sciences, Johns Hopkins Bloomberg School of Public Health, Baltimore, MD, United States

**Keywords:** myocarditis, endocrine disruptor, bisphenol A, estrogen receptor, mast cells

## Abstract

Myocarditis is an inflammatory heart disease that leads to dilated cardiomyopathy (DCM) and heart failure. Sex hormones play an important role in the development of myocarditis with testosterone driving disease in males and estrogen being cardioprotective in females. The human population is widely exposed to the endocrine disruptor bisphenol A (BPA) from plastics such as water bottles, plastic food containers, copy paper, and receipts. Several clinical and numerous animal studies have found an association between elevated BPA levels and cardiovascular disease. A recent report found elevated levels of BPA in the serum of patients with DCM compared to healthy controls. In this study we examined whether exposure to BPA for 2 weeks prior to viral infection and leading up to myocarditis at day 10 altered inflammation in female BALB/c mice housed in standard plastic cages/water bottles with soy-free food and bedding. We found that a human relevant dose of BPA (25 μg/L) in drinking water, with an estimated exposure of 5 μg BPA/kg BW, significantly increased myocarditis and pericarditis compared to control water without altering viral genome levels in the heart. BPA exposure activated ERα and ERβ in the spleen 24 h after infection and phosphorylated ERα and ERβ during myocarditis, but decreased ERα and increased ERβ mRNA in the heart as measured by qRT-PCR. Exposure to BPA significantly increased CD4^+^ T cells, IFNγ, IL-17A, TLR4, caspase-1, and IL-1β in the heart. BPA exposure also increased cardiac fibrosis compared to controls. Mast cells, which are associated with cardiac remodeling, were found to increase in number and degranulation, particularly along the pericardium. Interestingly, plastic caging/water bottle exposure alone led to increased mast cell numbers, pericardial degranulation and fibrosis in female BALB/c mice compared to animals housed in glass cages/water bottles with soy-free food and bedding. These data suggest that BPA exposure may increase the risk of developing myocarditis after a viral infection in women.

## Introduction

Myocarditis is an inflammatory heart disease that can lead to dilated cardiomyopathy (DCM) and heart failure and is most often caused by viral infections such as coxsackievirus B3 (CVB3) (1–3). We recently reported that cardiac inflammation during viral myocarditis in mice is increased by testosterone and reduced by 17β-estradiol, and the sex ratio in patients with myocarditis in the study was 3.5:1 male to female (4). Additionally, men with myocarditis are more likely to develop cardiac fibrosis than women and progress to DCM and heart failure (5, 6).

Estrogen receptors (ERs) are located on/in immune cells, cardiomyocytes, endothelial cells, and cardiac fibroblasts (7–9). ERα is believed to mediate most of the cardioprotective effects of estrogen in women and female mice (7). ERα has been found to protect against CVB3 myocarditis by increasing disease in ERα knockout mice while infected male mice treated with the ERα agonist propylpyrazoletriol were protected (10, 11). ERβ protects male and female mice from hypertrophy induced using transverse aortic constriction (TAC) by decreasing genes associated with mitochondrial damage, especially in males (12). However, ERβ has also been found to increase collagen synthesis from cardiac fibroblasts in male and female mice, while ERα decreases collagen synthesis (13–15). In CVB3 myocarditis, ERβ signaling was found to promote myocarditis in male or female mice treated with the ERβ agonist diarylpropionitrile (10, 11). Little is known about the effect of estrogen-related receptor (ERR)γ signaling on/in immune cells or on cardiac physiology or disease (16). Since CVB3 myocarditis is influenced by sex hormones and ERs, endocrine disruptors could play a role in the development and severity of disease.

People of all ages have detectable levels of bisphenol A (BPA) or its metabolized products in their body fluids (17, 18). BPA, an endocrine disruptor, is known to be found in items such as plastic water bottles, plastic food containers, the lining of cans, on thermal receipts, and photocopy paper (19–22). BPA has been found in nearly all patients when assessed in urine or blood, but the actual effect on heath and disease is largely unknown (18).

There have been studies assessing the pharmacokinetics and metabolism of BPA in humans and mice including following oral exposure. The half-life of BPA is 6 h and it is primarily metabolized to the glucuronide form prior to excretion (23). BPA has other metabolites that make up a smaller portion of its metabolism (23). BPA is metabolized to BPA glucuronide by UDP-glucuronosyltransferase and by sulfotransferase to BPA sulfate to a lesser degree (24). BPA is excreted in both the feces and urine with unconjugated BPA primarily excreted in feces whereas metabolized BPA is more predominantly excreted in the urine (23). Studies have also determined that the exposure route of BPA influences its pharmacokinetics and the clinical relevance of animal studies. Oral exposure of BPA has been found to more closely match levels found in humans compared to subcutaneous injection or bolus gavage routes (25). Recent studies have concluded that the pharmacokinetics of oral exposure to BPA is more similar between mice and humans than originally thought (26). Serum levels of BPA in mice vary greatly depending on the exposure route, with an oral exposure route being the most clinically relevant (27).

BPA can act as an estrogen agonist when it binds ERs including ERα, ERβ, and ERRγ but BPA has also been found to act on the androgen receptor (AR) (28, 29). BPA can bind to the AR where it acts in an anti-androgenic manner, but the AR is not able to bind to the androgen response element when BPA is bound instead of androgen (30). BPA preferentially binds ERβ over ERα but the metabolized forms of BPA, such as BPA glucuronide, do not bind the ERs (28). Epidemiological and animal data indicate that increased exposure to BPA worsens cardiovascular diseases including hypertension (31–33), atherosclerosis (34–36), myocardial infarct (37), arrhythmias (13, 14), and DCM (38). To our knowledge, no one has examined the role of BPA on myocarditis. In this study we examined whether BPA exposure in drinking water could alter CVB3 myocarditis in adult female BALB/c mice housed in plastic cages. Different doses of BPA were dissolved in drinking water for 2 weeks prior to intraperitoneal (ip) injection with CVB3 and self-tissue to induce autoimmune myocarditis and exposure continued until harvest at day 10 post-infection (pi). In order to determine the possible effect of exposure of mice to BPA or other plastics/ chemicals leached from the plastic cages, we compared the effect of plastic vs. glass cages/housing on myocarditis in separate experiments. Our autoimmune model of CVB3 myocarditis has the advantage of being a highly translatable animal model where the disease time-course, pathology, severity, sex differences, biomarkers and progression to DCM closely match patients (4, 39), providing a good animal model to test whether BPA is able to alter cardiac inflammation and the severity of disease.

## Materials and Methods

### Animal Care Ethics Statement

Mice were used in strict accordance with the recommendations in the Guide for the Care and Use of Laboratory Animals of the National Institutes of Health. Mice were maintained under pathogen-free conditions in the animal facility at the Johns Hopkins School of Medicine and at Mayo Clinic Florida, and approval obtained from the Animal Care and Use Committee at Johns Hopkins University and Mayo Clinic Florida for all procedures. Mice were sacrificed according to the Guide for the Care and Use of Laboratory Animals of the National Institutes of Health.

### CVB3-Induced Autoimmune Myocarditis Model

Female BALB/c (stock #651) 5 week old adult mice were obtained from the Jackson Laboratory (Bar Harbor, ME). Mice were maintained under pathogen-free conditions in the animal facility at the Johns Hopkins School of Medicine or the Mayo Clinic Florida animal facility. The number of mice used in individual experiments are listed in the text or figure legends. At 8 weeks of age mice were inoculated intraperitoneally (ip) with 10^3^ plaque forming units (PFU) of heart-passaged stock of CVB3 that contained cardiac self-tissue on day 0 and acute myocarditis examined at day 10 pi, as previously described (40). CVB3 (Nancy strain) was originally obtained from the American Type Culture Collection (ATCC; Manassas, VA) and grown in Vero cells (ATCC), as previously described (40).

### Bisphenol A and Housing

Mice arrived from Jackson Labs at 5 weeks of age and were placed in plastic cages/plastic water bottles for BPA exposure experiments or glass cages/glass water bottles for control experiments. All experiments used bedding (Envigo-Tekland, 7990.BG) and food (Envigo-Tekland, 2020X) from Envigo (Minneapolis, MN) that were free of soy and phytoestrogens to exclude other naturally occurring estrogenic compounds.

Varying doses of BPA were given to 6 week old mice dissolved in drinking water for 2 weeks prior to inoculation ip with CVB3 containing cardiac self-tissue. BPA exposure was continued from day 0 of viral infection until harvest at day 10 pi. At harvest heart tissue was divided in half and ½ heart used for histology, qRT-PCR, ELISA or western blot. Separate experiments were conducted in order to obtain all endpoints. Estimated intake of BPA for mice in drinking water was based on Jenkins et al. (41) who found that a 20 g mouse drinks an estimated 4 mL of water a day (Table 1). Jenkins et al. and Cagen et al. previously reported that BPA at these doses in water is stable for 1 week (41, 42). For this reason, as well as to provide the mice with fresh water, BPA water was replaced each week of the experiment.

**Table 1:** Bisphenol A (BPA) doses^a^.

**Treatment (μg BPA/L)**	**Estimated intake (μg BPA/kg BW)**	**Human exposure level**
0	0	Control
2.5	0.5	Human relevant exposure
25	5	High human relevant exposure
250	50	EPA reference dose

a *(41)*.

The doses of BPA that were administered were 2.5, 25, and 250 μg BPA/L in drinking water, which is equivalent to an estimated intake of 0.5, 5, and 50 μg BPA/kg body weight (BW), respectively, based on predicted daily exposure levels in the human population [(41); Table 1]. At the time of the development of this project Jenkins et al. was the only study available that assessed the effect of BPA in a mouse model using oral exposure with a clinically relevant dose of BPA in the drinking water. The EPA reference dose was calculated using a safety factor of 1,000x the lowest observable adverse effect level (LOAEL) (43). The EPA reference dose is defined as an estimate of the daily exposure to a susceptible individual without an appreciable risk of deleterious effects during a lifetime.

### Histology

Mouse hearts were cut longitudinally and fixed in 10% phosphate-buffered formalin and embedded in paraffin for histological analysis. Five micrometer sections were stained with hematoxylin and eosin (H&E) to detect inflammation, Masson's trichrome and picrosirius red to detect collagen or toluidine blue to detect mast cell granules. Myocarditis, pericarditis and fibrosis were assessed as the percentage of the heart with inflammation or fibrosis compared to the overall size of the heart section using a microscope eyepiece grid, as previously (44). Sections were scored by at least two individuals blinded to the treatment group.

### Quantitative Real-Time PCR

At harvest spleen or half heart was collected and stored at −80°C for RNA isolation. Spleens and hearts were homogenized and lysed using a Tissuelyser (Qiagen) with 7 mm stainless steel beads in RTL buffer with 0.5% DX buffer to reduce foam (Hilden, Germany). The homogenate was placed in an automated RNA isolation and purification instrument, QIAcube, with reagents for RNase Easy Mini Kit with a DNase step for spleen and cells or RNase Easy Fibrous Mini Kit including a DNase and proteinase K step for heart tissue (Qiagen). Spleen RNA was eluted into 30 μL and heart RNA into 60 μL RNase free water (Qiagen). RNA quantification was determined in μg/μL using NanoDrop (Thermo Scientific, Waltham, MA).

### Cell Isolation

Cardiac tissue was dissociated using enzyme buffer mixtures from the Miltenyi Biotec Mouse and Rat Heart Dissociation kit (#130-098-373) in gentleMACS C tubes (#130-098-237; 130-096-334) according to manufacturer's instructions. CD45 cells were isolated from the heart using the Miltenyi Biotec “LS” bead capture system (CD45 Ly-5 MicroBeads rat IgG2b, clone 30F11.1). We confirmed that the CD45 population had a purity of 94% while the cardiac/cardiomyocyte fraction was 97% pure using qRT-PCR for the markers CD45 and Myl2, respectively.

### qRT-PCR

Total messenger RNA (mRNA) from mouse spleens, hearts or heart cell isolates was assessed by quantitative real time (qRT)-PCR using Assay-on-Demand primers and probe sets and the ABI 7000 Taqman System from Applied Biosystems (Foster City, CA) after RNA was converted to cDNA using High Capacity cDNA Reverse Transcriptase Kit (Applied Biosystems), as previously described (44). Data are shown as relative gene expression (RGE) normalized to the housekeeping gene hypoxanthine phosphoribosyltransferase 1 (Hprt).

### Measurement of CVB3 Genome Levels by qRT-PCR

Probe sets to detect CVB3 in the heart were developed by Antoniak et al. and obtained from Integrated DNA Technologies (Coralville, IA) (45). Probe sets: CVB3 forward, 5′-CCCTGAATGCGGCTAATCC-3′; CVB3 reverse, 5′-ATTGTCACCATAAGCAGCCA-3′; CVB3 probe, 5′-FAM-TGCAGCGGAACCG-TAMRA-3′.

### ELISA

Mouse hearts were homogenized at 10% w/v in 2% minimal essential medium for use in ELISAs (R&D Systems, Minneapolis, MN), as previously described (46).

### Western Blot

Heart tissues were dissected, snap frozen on dry ice and stored at −80°C. Tissues were lysed by homogenizing using mechanical cell disperser with RIPA buffer (Santa Cruz Biotechnology) and protease/phosphate inhibitor cocktail (BioRad) to obtain proteins from membrane, cytoplasm and nucleus. Extracted proteins were separated on a Criterion XT precast bis-tris 4–12% gel (BioRad) then transferred onto nitrocellulose membrane (BioRad). Samples were probed with primary antibodies (Abcam) ERα (mouse IgG_1_ monoclonal, H226, 1:200), phospho-ERα (rat polyclonal IgG, Serine 118, 1:200), ERβ (mouse monoclonal IgG_2a_, 1531 1:200), phospho-ERβ (rat polyclonal IgG, Serine 87, 1:200), and normalized to Hprt (goat polyclonal IgG, N-15, 1:200). The molecular weight of the proteins of interest were: Hprt−23 kDa, ERα/ pERα-66 kDa, and ERβ/pERβ-56 kDa. The phosphorylation of the ER does not add additional molecular weight when assessing via western blot. Species-specific secondary antibodies were conjugated to horseradish peroxidase (HRP). Protein concentrations were normalized to the housekeeping gene (Hprt) and experimental groups were compared using densitometric analyses (Image J).

### Statistical Analysis

Normally distributed data comparing two groups were analyzed using a 1- or 2-tailed Student's *t*-test. The Mann-Whitney rank sum test was used to evaluate non-parametric data. When comparing more than two groups 1-way or 2-way ANOVA was performed followed by multiple comparison analysis (Dunnett's multiple comparisons test or Tukey's multiple comparison, respectively) with each group compared to the corresponding control group. Data are expressed as mean ± SEM. A value of *p* < 0.05 was considered significant.

## Results

### Disease Development in Response to BPA

#### BPA Exposure in Drinking Water Increases Viral Myocarditis in Female BALB/c Mice Housed in Plastic Cages

**Figure 1:**
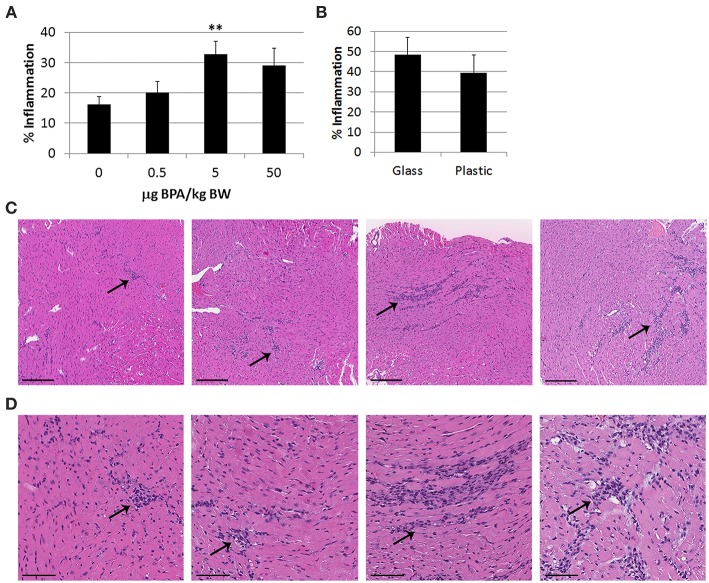
BPA exposure in drinking water increases viral myocarditis in female BALB/c mice housed in plastic cages. **(A)** Female BALB/c mice housed in plastic cages with no soy in food or bedding were given 0, 0.5, 5, and 50 μg BPA/kg BW BPA in drinking water for 2 weeks and then injected ip with 10^3^ PFU of CVB3 on day 0 and exposure continued until harvest for myocarditis at day 10 pi. Myocarditis was assessed as % inflammation in the heart compared to the overall size of the heart section by histology using an eyepiece grid. Data show the mean ±SEM (0 μg BPA/kg *n* = 10, 0.5 μg BPA/kg *n* = 9, 5 μg BPA/kg *n* = 9, 50 μg BPA/kg *n* = 10). One-way ANOVA found a significant difference between all groups (*p* = 0.01) and 0 vs. 5 μg BPA/kg BW (**p < 0.01). **(B)** Female BALB/c mice were housed in glass or plastic cages with glass or plastic water bottles, respectively, and no soy in food or bedding for 2 weeks prior to ip infection with CVB3 to induce myocarditis. The drinking water did not contain BPA. Mice were injected ip with 10^3^ PFU CVB3 ip on day 0 and hearts were harvested at day 10 pi during acute myocarditis. Data show the mean ± SEM (glass *n* = 10, plastic *n* = 10). Two-tailed Student's *t*-test found no significant difference between groups. **(C,D)** Representative H&E photos depict myocarditis at 0, 0.5, 5, and 50 μg BPA/kg BW at **(C)** magnification x100, scale bar 200 μm or **(D)** magnification x300, scale bar 70 μm, arrows indicate inflammatory foci.

To assess the effect of BPA exposure on CVB3-induced myocarditis, female adult BALB/c mice housed in traditional plastic cages were fed varying doses of BPA in drinking water *ad libitum* for 2 weeks prior to ip infection with CVB3 and heart tissues on day 0 until harvest at day 10 pi during myocarditis. Mice were provided soy-free bedding and food, because soy contains genistein which is estrogenic. We found that a high human relevant dose (5 μg BPA/kg BW) (*p* = 0.006) and EPA reference dose (50 μg BPA/kg BW) (*p* = 0.04) of BPA increased myocarditis compared to control water without BPA (0 BPA) using 2-tailed Student's *t*-test and one-way ANOVA (all doses *p* = 0.01) (Figure 1A). However, after adjusting for multiple comparisons only the 5 μg/kg BW dose of BPA was significantly different from the control water (*p* < 0.01).

Laboratory animals are traditionally housed in polycarbonate cages with polysulfone water bottles. Both of these plastics contain BPA which can leach from the plastic, especially when heated as occurs when cages are routinely autoclaved for sterilization (47). As cages age, more plastic can leach due to degradation of the surface (47). In order to determine whether BPA that may have leached from the plastic cages and plastic water bottles used in our experiments affected myocarditis, in separate experiments we compared glass cages and water bottles (no added BPA) to plastic cages and water bottles (possible BPA or other plastics/chemicals released due to leaching). We used soy-free bedding and food to remove the effect of these estrogenic compounds. The water bottles in glass and plastic cages contained normal water (i.e., did not contain additional BPA) throughout the experiment. Female BALB/c mice were infected with CVB3 ip on day 0 and myocarditis assessed at day 10 pi. We found that there was no significant difference between the level of myocardial inflammation (i.e., myocarditis) that developed in female BALB/c mice housed in plastic compared to glass cages (*p* = 0.50) (Figure 1B). Thus, BPA or other chemicals that leached from plastic cages and/or plastic water bottles was not able to increase myocarditis, indicating that BPA added to the drinking water was responsible for the increase in myocarditis over controls.

#### BPA Exposure in Drinking Water Does Not Alter Viral Levels in the Heart of Female BALB/c Mice Housed in Plastic Cages

We examined whether BPA exposure in drinking water altered CVB3 levels in the heart during myocarditis at day 10 pi in female mice housed in plastic cages. We found that BPA exposure did not significantly alter viral genome levels in the heart by qRT-PCR for any dose of BPA (0 BPA vs. 0.5 μg BPA/kg BW, *p* = 0.29; vs. 5 μg BPA/kg BW, *p* = 0.13; or vs. 50 μg BPA/kg BW, *p* = 0.41). There was also no difference in viral genome levels in female BALB/c mice with myocarditis that were housed in plastic vs. glass cages without addition of BPA to drinking water (*p* = 0.18). Thus, BPA exposure in drinking water did not alter viral replication, and increased myocarditis with BPA exposure was not due to increased viral replication.

#### BPA Exposure in Drinking Water Increases Pericarditis in Female BALB/c Mice Housed in Plastic Cages

**Figure 2:**
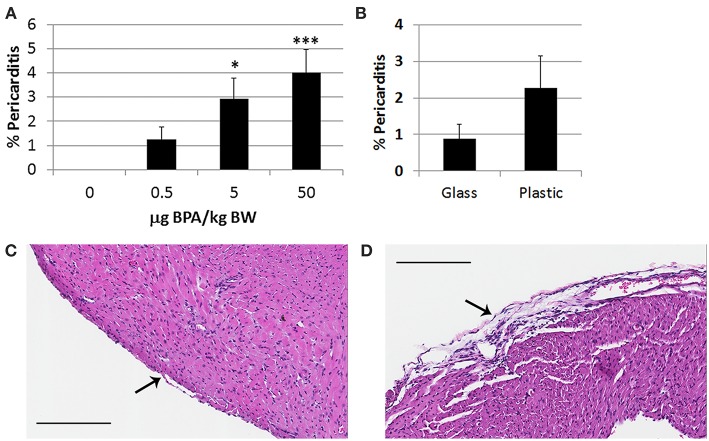
BPA exposure in drinking water increases pericarditis in female BALB/c mice housed in plastic cages. Female BALB/c mice housed in plastic cages with no soy in food or bedding were given increasing doses of BPA in drinking water for 2 weeks and injected with 10^3^ PFU CVB3 ip on day 0 and pericarditis examined at day 10 pi during acute myocarditis. BPA exposure continued from day 0 to 10 pi. Pericarditis was assessed as the % pericardial inflammation in the heart with H&E compared to the overall size of the heart section by histology using an eyepiece grid. **(A)** Data show the mean ± SEM (0 μg BPA/kg *n* = 10, 0.5 μg BPA/kg *n* = 9, 5 μg BPA/kg *n* = 9, 50 μg BPA/kg *n* = 10). One-way ANOVA found a significant difference existed between groups (*p* = 0.002). After controlling for multiple comparisons, the 5 and 50 μg BPA/kg BW groups were significantly different than 0 BPA control water (*p < 0.05 and ***p < 0.001, respectively). **(B)** Female BALB/c mice were housed in glass or plastic cages with glass or plastic water bottles and no soy in food or bedding for 2 weeks prior to ip infection with CVB3 to induce myocarditis. The drinking water did not contain BPA. Data show the mean ± SEM (glass *n* = 10, plastic *n* = 10). Two-tailed Student's *t*-test found no significant difference between groups. Representative photos depict **(C)** 0 BPA and **(D)** 50 μg BPA/kg BW, bar 200 μm, magnification 100x, arrows point to outer pericardial layer of the heart.

Pericarditis is common in patients with myocarditis and is observed in our animal model (48). We found that BPA exposure in drinking water of female BALB/c mice housed in plastic cages significantly increased pericarditis at day 10 pi (1-way ANOVA, *p* = 0.002) (Figure 2A). Controlling for multiple comparisons revealed that the 5 μg and 50 μg/kg BW doses of BPA significantly increased pericarditis at day 10 pi compared to control water (*p* < 0.05 and *p* < 0.001, respectively). When we examined whether BPA leached from plastic cages or water bottles could alter pericarditis compared to glass cages and water bottles we found that there was no significant difference between glass and plastic caging (*p* = 0.18) (Figure 2B). Normal pericardium consists of one layer of pericardial cells on the outer surface of the heart as depicted in Figure 2C. A representative photo of pericarditis that was promoted by exposure to BPA in drinking water is depicted in Figure 2D.

#### BPA Exposure in Drinking Water Increases Cardiac CD4^+^ T and Mast Cells in Mice Housed in Plastic Cages, Plastic Cages Alone Increase Mast Cells

Because myocarditis was significantly increased by the high human relevant dose of BPA (i.e., 5 μg BPA/kg BW) (Figure 1), all future analyses compared the 0 BPA to the 5 μg BPA/kg BW (5 BPA) groups.

In female BALB/c mice exposure to 5 μg BPA/kg BW significantly increased cardiac CD4^+^ T cells based on expression of CD4 levels (*p* = 0.03) and mast cells based on cKit/CD117 expression (*p* = 0.004) vs. 0 BPA controls (Table 2). We also examined expression of CD45, GR1, CD11b, F4/80, CD3, CD8, B220, and Foxp3, but found no significant difference between 0 BPA and 5 μg BPA/kg BW for any of the other immune cell markers (Table 2). An increase in CD4^+^ T cells and mast cells during myocarditis following BPA exposure is consistent with the increased myocardial and pericardial inflammation observed with histology for the 5 μg BPA/kg BW dose (Figures 1, 2). Interestingly, cardiac mast cells were increased by qRT-PCR based on cKit expression in plastic vs. glass cages in mice with CVB3 myocarditis (Table 3). No other immune cell population was significantly altered by exposure to plastic compared to glass cages (Table 3).

**Table 2:** Effect of BPA exposure in drinking water in mice housed in plastic cages on expression of immune cell markers in the heart during myocarditis using qRT-PCR.

**Cell marker**	**Description**	**0 BPA**^**a**^^**,**^ ^**b**^	**5 BPA**	***P*-value**
CD45	Total immune cells	4.3 ± 1.1	6.2 ± 1.8	0.43
GR1	Neutrophils	7.5 ± 0.7	7.4 ± 2.2	0.35
CD11b	Mac, neu, MC, DC	4.7 ± 0.8	3.1 ± 0.6	0.09
F4/80	Macrophages	4.7 ± 0.6	4.1 ± 0.7	0.47
CD3	All T cells	20.3 ± 8.3	4.4 ± 1.4	0.43
CD4	CD4^+^ T cells	2.8 ± 0.4	4.9 ± 1.1	0.03
CD8	CD8^+^ CTL cells	108.9 ± 58.6	6.3 ± 2.0	0.75
Foxp3	Regulatory T cells	20.3 ± 8.3	4.4 ± 1.4	0.43
B220	B cells	10.5 ± 3.9	5.4 ± 1.1	0.58
cKit	MC	76.6 ± 6.1	131.7 ± 20.5	0.004

a CLT, cytolytic T cells; DC, dendritic cells; Mac, macrophages; MC, mast cells; Neu, neutrophils.

b *0 BPA, control water without BPA; 5 BPA, high human relevant dose (5 μg/kg BW)*.

**Table 3:** Effect of plastic vs. glass housing on expression of immune cell markers in the heart during myocarditis using qRT-PCR.

**Cell marker**	**Description**	**Glass**	**Plastic**	***P*-value**
CD45	Total immune cells	8.72 ± 1.8	8.2 ± 2.2	0.87
CD11b	Mac^a^, neu, MC, DC	6.2 ± 1.0	5.5 ± 1.3	0.68
F4/80	Macrophages	6.8 ± 1.0	6.3 ± 1.4	0.77
CD3	All T cells	6.9 ± 1.6	6.3 ± 1.3	0.73
CD4	CD4^+^ T cells	10.1 ± 1.9	9.1 ± 2.5	0.77
CD8	CD8^+^ CTL cells	7.7 ± 1.1	7.1 ± 2.4	0.81
Foxp3	Regulatory T cells	2,073 ± 1,191	1,990 ± 627	0.95
CD19	B cells	5.9 ± 1.4	4.6 ± 1.5	0.53
cKit	Mast cells	1.9 ± 0.2	3.1 ± 0.4	0.04

a *CLT, cytolytic T cells; DC, dendritic cells; Mac, macrophages; MC, mast cells; Neu, neutrophils*.

#### BPA Exposure in Drinking Water Increases Cardiac Mast Cell Numbers and Pericardial Mast Cell Degranulation in Mice Housed in Plastic Cages

**Figure 3:**
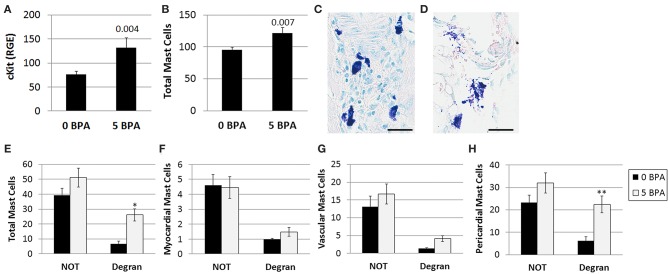
BPA increases the number of mast cells and degranulation of pericardial mast cells during viral myocarditis. Female BALB/c mice were given 0 or 5 μg/kg BW BPA (0 BPA vs. 5 BPA) in drinking water for 2 weeks and injected with 10^3^ PFU CVB3 ip on day 0. BPA exposure continued from day 0 to 10 pi and hearts were harvested at day 10 pi during acute myocarditis. **(A)** Relative gene expression (RGE) of mast cells (cKit/ CD117) vs. the housekeeping gene Hprt was analyzed in whole hearts by qRT-PCR at day 10 pi comparing 0 to 5 μg BPA/kg BW groups. (0 BPA *n* = 10, 5 BPA *n* = 8). Toluidine blue was used to detect mast cell granules by histology and the total numbers of mast cells in the heart section were normalized to the size of the heart using an eyepiece grid (50). **(B)** Data show the mean ± SEM using the Mann-Whitney rank test (0 BPA *n* = 20, 5 BPA *n* = 20). A representative photo of **(C)** non-degranulating (NOT) and **(D)** degranulating (Degran) mast cells. Mast cell granules stain dark purple, magnification 400x, scale bar 30 μm. **(E)** Data show the mean ± SEM of 10 mice/group. Two-way ANOVA found significant results for degranulation (*p* < 0.0001) and BPA (*p* = 0.0006). *p < 0.05 indicates *ad-hoc* analysis comparing 0 vs. 5 BPA for degranulating cells (Degran). **(F,G)** Two-way ANOVA found significant results for degranulation *p* = 0.0001, but not for BPA for **(F)** myocardial and **(G)** vascular MCs, *n* = 10/group. **(H)** In contrast, 2-way ANOVA found significant results for degranulation (*p* < 0.0002) and BPA (*p* = 0.0004) for pericardial mast cells. After Tukey's multiple comparison test, 5 BPA was found to significantly increase degranulation in pericardial mast cells compared to control water (**p < 0.01).

Previously, BPA exposure in culture was found to increase mast cell number and activation (49); however, the effect of BPA on cardiac mast cells during myocarditis was unknown. The effect of 5 μg BPA/kg BW exposure on cardiac mast cells during myocarditis at day 10 pi was assessed using qRT-PCR and toluidine blue staining of heart histology sections, which detects mast cell granules. We found that BPA exposure significantly increased the expression of the mast cell marker cKit by qRT-PCR in the heart (0 BPA 76.6 ± 6.1 vs. 5 BPA 131.7 ± 20.5, *p* = 0.004) (Figure 3A).We found that BPA exposure significantly increased the total number of mast cells in the heart by histology (*p* = 0.007) compared to 0 BPA control (Figure 3B). A representative photo of mast cells that are not degranulating are shown in Figure 3C and degranulating mast cells in Figure 3D.

By 2-way ANOVA we found that during myocarditis more mast cells were not degranulating compared to those that were degranulating (*p* < 0.0001) (Figure 3E), and BPA exposure in drinking water increased mast cell degranulation in the heart overall (2-way ANOVA, *p* = 0.0006) (Figure 3E). However, 5 μg BPA/kg BW did not increase the number of mast cells that were not degranulating, but significantly increased the number of degranulating mast cells during myocarditis (*p* < 0.05) compared to the 0 BPA group (Figure 3E).

Next we examined the number of degranulating vs. non-degranulating mast cells in different locations of the heart (i.e., myocardial, vascular, pericardial). We found that more myocardial mast cells were not degranulating than degranulating (*p* < 0.0001) (Figure 3F), but note the few number of mast cells found at this location. BPA did not increase the number of myocardial mast cells in the heart overall (*p* = 0.71) (Figure 3F). Two-way ANOVA and a Student's *t*-test (*p* = 0.09) comparison of 0 BPA to 5 BPA did not find a significant difference in mast cell degranulation in the myocardium. Similar to myocardial and overall mast cell numbers, there was no increase in the number of vessel-associated mast cells that were not degranulating vs. degranulating following BPA exposure (*p* = 0.11) (Figure 3G). There was an increase in vessel-associated mast cells that were degranulating after BPA exposure using Student's *t*-test (*p* = 0.005), but this difference was no longer significant by 2-way ANOVA (Figure 3G). Similar to other locations, more pericardial mast cells were not degranulating than degranulating (2-way ANOVA, *p* < 0.0002) (Figure 3H). In contrast to other locations, however, BPA exposure significantly increased degranulation of pericardial mast cells overall (2-way ANOVA, *p* = 0.0004) and in the degranulating subgroup (*p* < 0.01) compared to the 0 BPA controls (Figure 3H). Note that the largest numbers of mast cells in the heart during myocarditis are located along the pericardium.

We found other markers of mast cell activation were also increased in the heart during myocarditis by 5 BPA compared to control by qRT-PCR including IgE receptor-γ (0 BPA 1.7 ± 0.1 vs. 5 BPA 2.1 ± 0.0.3, *p* = 0.03) and the anaphylaxis receptor C3aR1 (0 BPA 2.3 ± 0.2 vs. 5 BPA 3.1 ± 0.3, *p* = 0.04).

#### Plastic Cages Increase Cardiac Mast Cell Numbers and Pericardial Mast Cell Degranulation

**Figure 4:**
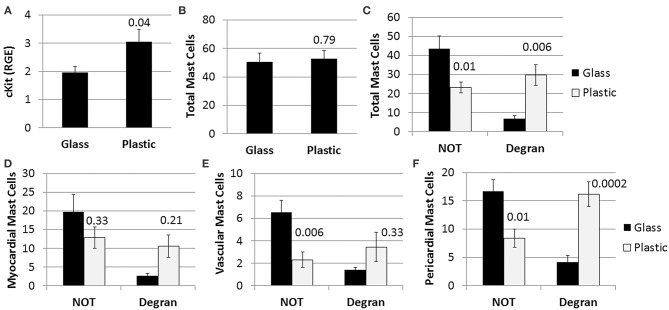
Plastic cages/water bottles, without the addition of BPA in water, increase mast cell numbers and pericardial degranulation in BALB/c females. Female BALB/c mice were housed in glass or plastic cages with glass or plastic water bottles and no soy in food or bedding for 3 weeks prior to ip infection with CVB3 to induce myocarditis. The drinking water did not contain BPA. Mice were injected ip with 10^3^ PFU of CVB3 ip on day 0 and hearts were harvested at day 10 pi during acute myocarditis. **(A)** Relative gene expression (RGE) of mast cells (cKit/ CD117) vs. the housekeeping gene Hprt was analyzed in whole hearts by qRT-PCR at day 10 pi comparing mice in glass vs. plastic cages (glass *n* = 10, plastic *n* = 10). **(B–F)** Toluidine blue was used to detect mast cell granules by histology and the total numbers of MCs in the heart section were normalized to the size of the heart using an eyepiece grid [(50); glass *n* = 10, plastic *n* = 10]. **(A,B)** Data show the mean ± SEM using the Mann-Whitney rank test of total mast cells (NOT and Degran). **(C–F)** Two-way ANOVA found significant results for degranulation (*p* < 0.0001) and BPA (*p* = 0.0006). *P*-values depict *ad-hoc* analyses comparing glass to plastic by Tukey's multiple comparison.

We found mast cell levels were significantly increased in the heart of female mice with myocarditis that were housed in plastic cages compared to those housed in glass cages based on the mast cell marker cKit (glass 2.0 ± 0.2 vs. plastic 3.1 ± 1.4, *p* = 0.04) (Figure 4A). Housing mice in plastic cages compared to glass cages did not alter the total number of mast cells assessed histologically by toluidine blue staining (*p* = 0.79) (Figure 4B). However, by 2-way ANOVA we found that during myocarditis more mast cells were not degranulating compared to those that were degranulating (*p* < 0.0001) for mice housed in glass cages, whereas a similar number of mast cells were degranulating vs. not degranulating for mice housed in plastic cages (*p* = 0.74) (Figure 4C). So comparing glass to plastic, overall more mast cells were degranulating in the hearts of mice housed in plastic cages than in glass cages (*p* = 0.006) (Figure 4C).

Examining the number of degranulating vs. non-degranulating mast cells in different locations of the heart (i.e., myocardial, vascular, pericardial), we found that more mast cells located in the myocardium were not degranulating compared to those that were degranulating by 2-way ANOVA (*p* < 0.0008) for mice housed in glass cages, whereas a similar number of mast cells were degranulating vs. not degranulating for mice housed in plastic cages (*p* = 0.94) (Figure 4D). Additionally, there was no significant difference in the number of myocardial mast cells that were degranulating in glass vs. plastic cages (*p* = 0.21). Similarly, most mast cells associated with vessels were not degranulating in the heart of mice housed in glass cages (*p* = 0.0006), and a similar number of vascular mast cells were degranulating vs. not degranulating in plastic cages (*p* = 0.78) (Figure 4E). There was no significant difference in the number of vessel-associated mast cells that were degranulating vs. not degranulating comparing glass to plastic cages (*p* = 0.33). Similar to myocardial and vascular mast cells, mast cells along the pericardium were mainly not degranulating if mice were housed in glass cages (*p* = 0.0001) (Figure 4F). In contrast to mast cells in the other locations, more pericardial mast cells were degranulating than not degranulating for mice housed in plastic cages (*p* = 0.02), and more pericardial mast cells were degranulating when mice were housed in plastic compared to glass cages (*p* = 0.0002) (Figure 4F). Thus, the elevated cKit levels detected by PCR from mice housed in plastic cages corroborate the increased number of pericardial degranulating mast cells detected in the heart using histology. Additionally, BPA, other plastics and/or other chemicals that are leaching from plastic cages/plastic water bottles are causing the increase in number and pericardial degranulation of mast cells observed in the experiments where mice are exposed to BPA in drinking water (Figure 3).

### BPA Effect on ERs in the Spleen and Heart

#### ERα, ERβ, and AR Are More Highly Expressed on Cardiac Tissue Than Immune Cells During Viral Myocarditis in Mice Housed in Plastic Cages

To determine whether immune cells (i.e., CD45^+^ cells) or cardiac tissue (i.e., cardiomyocytes, fibroblasts, endothelial cells) expressed more ERs or AR, we isolated these two cell populations from the heart during myocarditis in female BALB/c mice that were housed in plastic cages without soy in their food or bedding and no BPA added to their drinking water. We found that cardiac tissue had significantly higher expression of ERα (CD45 2.7 ± 0.4 vs. cardiac 17.9 ± 5.0, *p* = 1.3 × 10^−5^), ERβ (CD45 0.0 vs. cardiac 2.8 ± 1.2, *p* = 0.007), and AR (CD45 3.0 ± 0.6 vs. cardiac 42.2 ± 7.4, *p* = 1.3 × 10^−5^) compared to immune cells normalized to Hprt using qRT-PCR (data not shown). There was no significant difference between the two populations for ERRγ expression after normalization (CD45 20.8 ± 7.0 vs. cardiac 19.8 ± 8.9, *p* = 0.76).

#### BPA Exposure in Drinking Water Increases ERα and ERβ Expression at 24 h pi in the Spleen in Mice Housed in Plastic Cages

**Figure 5:**
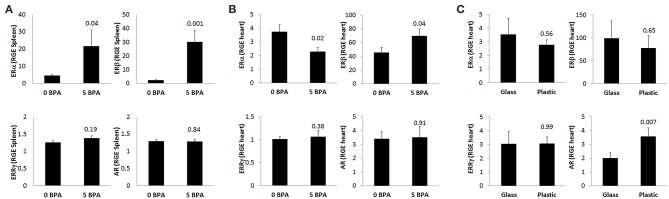
BPA exposure in drinking water increases splenic ERα/ERβ, decreases cardiac ERα but increases ERβ during myocarditis, while plastic cages increase cardiac AR during myocarditis. Relative gene expression (RGE) of ERα, ERβ, ERRγ, and AR vs. the housekeeping gene Hprt were analyzed in **(A)** spleen at 24 h (0 BPA *n* = 10, 5 BPA *n* = 10), **(B)** whole hearts at day 10 pi by qRT-PCR comparing 0–5 μg BPA/kg BW (0 BPA *n* = 10, 5 BPA *n* = 8) or **(C)** whole hearts at day 10 pi by qRT-PCR comparing glass vs. plastic caging (glass *n* = 10, plastic *n* = 10). Data show the mean ± SEM using a two-tailed Student's *t* or Mann-Whitney rank test.

To assess whether BPA exposure altered ER expression on/in splenic immune cells during the innate immune response to CVB3 infection, we exposed mice for 2 weeks prior to ip injection with CVB3 on day 0 to 5 μg BPA/kg BW (5 BPA) in drinking water and examined ERα, ERβ, ERRγ, and AR expression by qRT-PCR in the spleen at 24 h pi compared to 0 BPA (we continued BPA exposure until harvest). We found that BPA exposure significantly increased ERα (0 BPA 4.5 ± 0.5 vs. 5 BPA 21.6 ± 9.4, *p* = 0.04) and ERβ (0 BPA 2.2 ± 0.4 vs. 5 BPA 30.0 ± 8.5, *p* = 0.001) expression in the spleen, but had no significant effect on ERRγ (0 BPA 1.3 ± 0.1 vs. 5 BPA 1.4 ± 0.1, *p* = 0.19) or the AR (0 BPA 1.3 ± 0.1 vs. 5 BPA 1.3 ± 0.1, *p* = 0.84) compared to 0 BPA (Figure 5A, Table 4).

**Table 4:** Effect of BPA exposure in drinking water in mouse housed in plastic cages on expression of steroid hormones in spleen and heart using qRT-PCR.

**Cell marker**	**Description**	**0 BPA**^**a**^^**,**^ ^**b**^	**5 BPA**	***P*-value**
**24 hr spleen**				
ERα	Estrogen receptor alpha	4.5 ± 0.5	22 ± 9.4	0.04
ERβ	Estrogen receptor beta	2.2 ± 0.4	30 ± 8.5	0.001
ERRγ	Estrogen related receptor gamma	1.3 ± 0.1	1.4 ± 0.1	0.19
AR	Androgen receptor	1.3 ± 0.1	1.3 ± 0.1	0.84
**d10 heart**				
ERα	Estrogen receptor alpha	3.7 ± 0.5	2.3 ± 0.3	0.02
ERβ	Estrogen receptor beta	46 ± 7.4	70 ± 10	0.04
ERRγ	Estrogen related receptor gamma	1.0 ± 0.1	1.1 ± 0.1	0.38
AR	Androgen receptor	3.4 ± 0.5	3.5 ± 0.8	0.91

a AR, androgen receptor; d, day; ERα, estrogen receptor alpha; ERβ, estrogen receptor beta; ERRγ, estrogen related receptor gamma; hr, hour.

b *0 BPA, control water without BPA; 5 BPA, high human relevant dose (5 μg/kg BW)*.

#### BPA Exposure in Drinking Water Decreases ERα and Increases ERβ Expression in the Heart During Viral Myocarditis in Mice Housed in Plastic Cages

BPA binds to ERα and ERβ at a 10,000-fold lower affinity than natural estrogen, but binds ERRγ with a higher affinity (16). Therefore, exposure to BPA has the potential to alter myocarditis. BPA has been found to be able to bind to the AR directly (30), and one study found that BPA can act as both an agonist and antagonist for the AR (51). In this study, we found that exposure to 5 μg BPA/kg BW decreased the mRNA expression of ERα in the heart during CVB3 myocarditis (*p* = 0.02) and increased the expression of ERβ (*p* = 0.04), but had no significant effect on ERRγ (*p* = 0.38) or the androgen receptor (AR) (*p* = 0.91) (Figure 5B, Table 4). Based on our finding that BPA exposure increased acute myocarditis in female BALB/c mice (Figure 1), these data suggest that ERα signaling reduces while ERβ signaling increases our model of autoimmune CVB3 myocarditis- confirming the role for these receptors that have been shown previously by Huber et al. using a different model of CVB3 myocarditis (10). These findings also suggest that BPA mediates its effect by altering ERα and ERβ expression levels in the heart and/or on cardiac inflammation.

#### BPA Exposure in Drinking Water Activates Cardiac ERα and ERβ During Viral Myocarditis in Mice Housed in Plastic Cages

**Figure 6:**
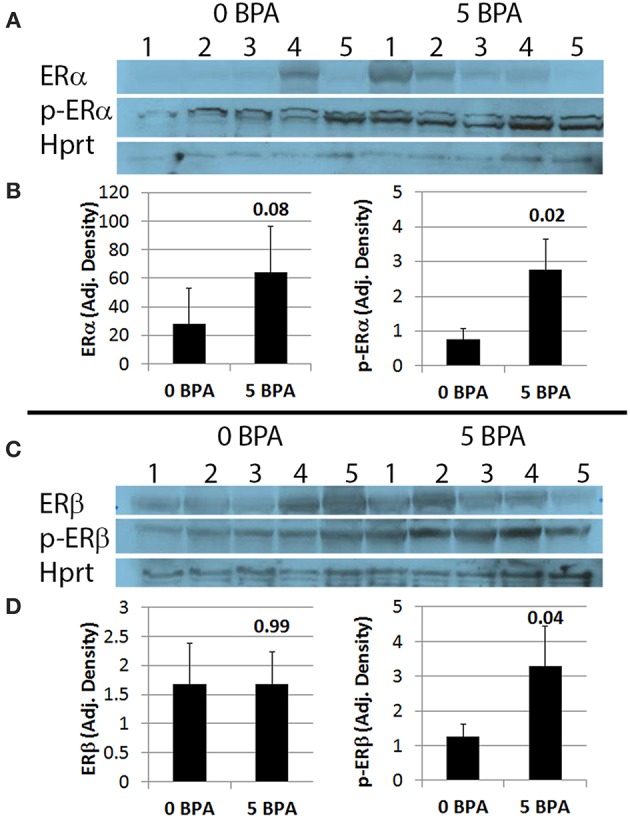
BPA exposure in drinking water significantly increases phosphorylation of ERα (p-ERα) and ERβ (p-ERβ) in the heart during myocarditis. **(A)** Western blot images for ERα, p-ERα and Hprt. One to five represent individual samples from hearts with myocarditis. **(B)** Quantitation of western blot data for ERα (left) and p-ERα (right) from hearts adjusted to Hprt levels. Data show the mean ± SEM using a two-tailed Mann-Whitney rank test with 5 mice/group. **(C)** Western blot images from hearts for ERβ, p-ERβ, and Hprt. One to five represent individual samples. **(D)** Quantitation of western blot data for ERβ (left) and p-ERβ (right) adjusted to Hprt levels. Data show the mean ± SEM using a one-tailed Mann-Whitney rank test with 5 mice/group.

Activation of ERs requires phosphorylation (52). We examined whether BPA altered activation of ERα and/or ERβ by determining protein levels of the phosphorylated form of the receptors by western blot. We found that BPA exposure in drinking water did not significantly alter total protein levels of ERα (Figures 6A,B) or ERβ (Figures 6C,D) in the heart during myocarditis, but significantly increased phosphorylated-ERα (p-ERα) (Figures 6A,B) and phosphorylated-ERβ (p-ERβ) (Figures 6C,D) in mice exposed to 5 μg BPA/kg BW. Thus, BPA exposure activated cardiac ERα and ERβ in the heart during myocarditis.

#### Plastic Cages Increase AR Expression in the Heart During Myocarditis

Next we determined ER and AR expression in the heart using qRT-PCR of female mice with myocarditis housed in glass vs. plastic cages with food and bedding that did not contain soy. We found that plastic cages significantly increased AR (*p* = 0.007) expression in females during acute myocarditis compared to glass cages, but did not significantly alter ER expression in the heart (Figure 5C, Table 5).

**Table 5:** Effect of plastic vs. glass caging on expression of steroid hormones in the heart using qRT-PCR.

**Cell marker**	**Description**^**a**^	**Glass**	**Plastic**	***P*-value**
**d10 heart**				
ERα	Estrogen receptor alpha	3.5 ± 1.2	2.8 ± 0.4	0.56
ERβ	Estrogen receptor beta	98 ± 38	77 ± 27	0.65
ERRγ	Estrogen related receptor gamma	3.1 ± 0.9	3.1 ± 0.5	0.99
AR	Androgen receptor	2.0 ± 0.4	3.6 ± 0.6	0.007

a *d, day; ERα, estrogen receptor alpha; ERβ, estrogen receptor beta*.

### BPA Effect on Inflammatory Mediators

#### BPA Exposure in Drinking Water Significantly Increases Cardiac IFNγ and IL-17A in Mice Housed in Plastic Cages

**Figure 7:**
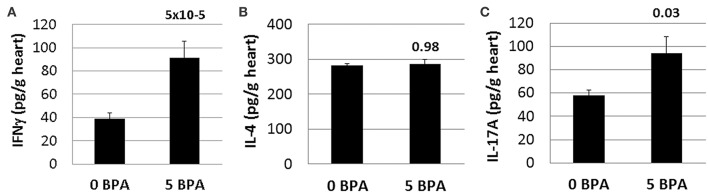
Cardiac IFNγ and IL-17A levels significantly increased by BPA exposure in drinking water. Hearts were harvested at day 10 pi and homogenized and the supernatant used to measure **(A)** IFNγ, **(B)** IL-4, and **(C)** IL-17A levels in the heart by ELISA. Data show the mean ± SEM using a one-tailed (IL-17A) or 2-tailed Student's *t* (IFNγ and IL-4) or Mann-Whitney rank test with 10 mice/group.

BPA exposure has been shown to increase both Th1 and Th2 immune responses in various inflammatory animal models, like the OVA model of asthma for example (53). Because CD4^+^ T cells were increased by BPA exposure in drinking water (Table 2), we examined whether IFNγ, IL-4, and/or IL-17A cytokine levels were altered in the heart during acute CVB3 myocarditis following BPA exposure. Changes in these cytokines are often used to indicate Th1, Th2, and/or Th17 responses, respectively (46). We found that IFNγ (0 BPA 39.2 ± 4.5 vs. 5 BPA 91.4 ± 14.3, *p* = 5 × 10^−5^) (Figure 7A) and IL-17A (0 BPA 57.8 ± 9.2 vs. 5 BPA 94.0 ± 15.5, *p* = 0.03) (Figure 7C) were significantly increased in the heart by exposure to 5 μg BPA/kg BW during myocarditis, but IL-4 levels were not significantly altered (0 BPA 282.5 ± 85.0 vs. 5 BPA 385.9 ± 72.1, *p* = 0.98) (Figure 7B).

#### BPA Exposure in Drinking Water Increases TLR4, Caspase-1, and IL-1β in the Heart During Myocarditis in Mice Housed in Plastic Cages

**Figure 8:**
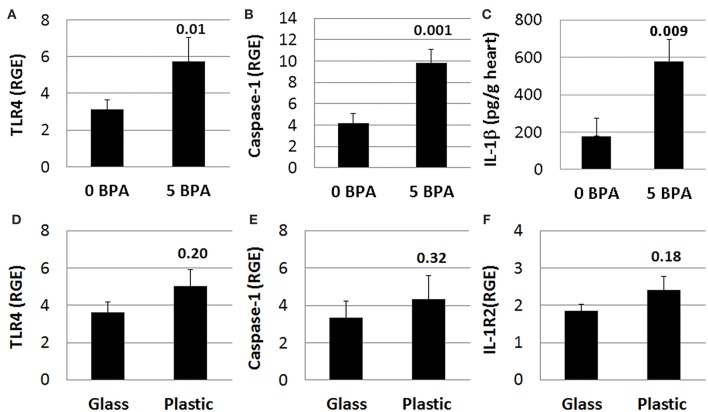
BPA exposure in drinking water activates TLR4 pathway during myocarditis. **(A–C)** BPA exposure in drinking water, **(D–F)** plastic vs. glass caging (no BPA added to water). Relative gene expression (RGE) of genes vs. the housekeeping control Hprt for **(A)** TLR4 (0 BPA *n* = 10, 5 BPA *n* = 7) and **(B)** caspase-1 (0 BPA *n* = 10, 5 BPA *n* = 9) were examined in whole hearts by qRT-PCR at day 10 pi comparing 0–5 μg BPA/kg BW. **(C)** IL-1β protein levels were determined using ELISA from homogenized whole heart supernatants comparing 0 to 5 μg BPA/kg BW (0 BPA *n* = 8, 5 BPA *n* = 10). RGE of genes vs. the housekeeping control Hprt for **(D)** TLR4 (glass *n* = 10, plastic *n* = 10), **(E)** caspase-1 (glass *n* = 10, plastic *n* = 10), and **(F)** IL-1R2 (glass *n* = 10, plastic *n* = 10) were examined in whole hearts by qRT-PCR at day 10 pi comparing glass to plastic. Data show the mean ± SEM using a two-tailed Student's *t* or Mann-Whitney rank test.

We previously demonstrated that components of IL-1R-mediated signaling (i.e., TLR4, caspase-1, IL-1β, and IL-18) are upregulated in the heart during viral myocarditis in male mice and that testosterone elevates this pathway on mast cells and macrophages during the innate and adaptive immune response to CVB3 infection in our model (44, 46). In this study we found that 5 μg BPA/kg BW exposure significantly increased expression of this pathway in the heart during acute CVB3 myocarditis in female BALB/c mice. We found that TLR4 (0 BPA 3.1 ± 0.5 vs. 5 BPA 5.7 ± 1.3, *p* = 0.01), caspase-1 (0 BPA 4.2 ± 0.9 vs. 5 BPA 9.8 ± 1.3, *p* = 0.001), and IL-1β (0 BPA 178.7 ± 97.3 vs. 5 BPA 577.9 ± 116.3, *p* = 0.009) levels were significantly increased by BPA exposure in drinking water compared to control water (Figures 8A–C). In contrast, BPA that may have leached from plastic cages and water bottles did not significantly alter TLR4 (glass 3.6 ± 0.6 vs. plastic 5.0 ± 0.9, *p* = 0.20), caspase-1 (glass 3.3 ± 0.7 vs. plastic 4.3 ± 0.7, *p* = 0.32) or IL-1R2 (glass 1.9 ± 0.2 vs. plastic 2.4 ± 0.4, *p* = 0.18) in the heart (Figures 8D–F) indicating that BPA exposure in drinking water led to elevated TLR4 signaling rather than plastic caging alone.

#### BPA Exposure in Drinking Water Increases Pericardial, Myocardial, and Vascular Fibrosis

**Figure 9:**
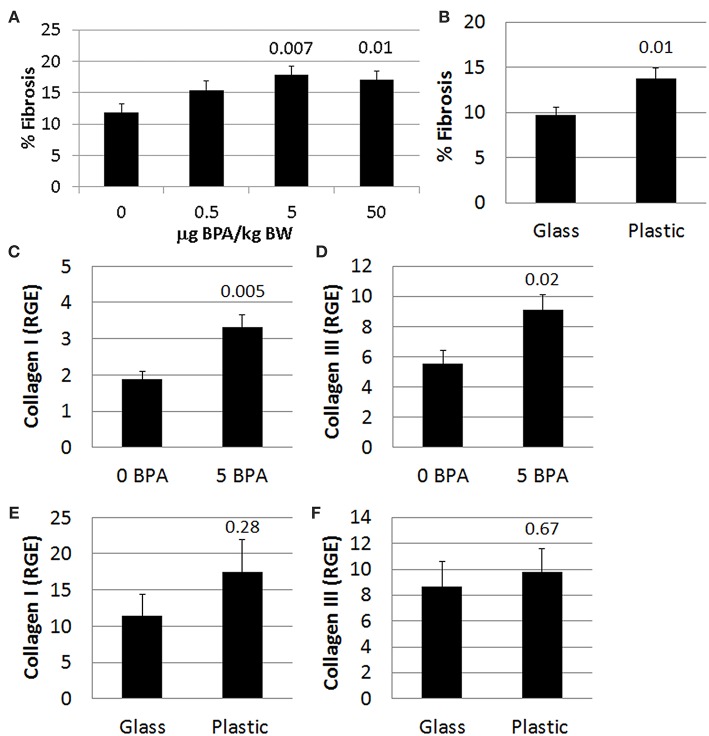
BPA exposure increased fibrosis. Female BALB/c mice were given 0, 0.5, 5, and 50 μg BPA/kg BW in drinking water for 2 weeks and then injected ip with 10^3^ PFU of CVB3 on day 0 and exposure continued until harvest for myocarditis at day 10 pi. Fibrosis in the heart was assessed using Masson's trichrome blue to detect collagen deposition, which stains bright blue. **(A,B)** Quantification of cardiac fibrosis was calculated histologically as % collagen staining blue in the heart normalized to the overall size of the heart section using an eyepiece grid. Data show the mean ± SEM. **(A)** comparing BPA exposed mice to mice given no BPA in water (0 μg BPA/kg *n* = 10, 0.5 μg BPA/kg *n* = 9, 5 μg BPA/kg *n* = 9, 50 μg BPA/kg *n* = 10). One-way ANOVA found a significant difference existed between groups (*p* = 0.04). After controlling for multiple comparisons (Dunnett's multiple comparisons), the 5 and 50 μg BPA/kg BW groups were significantly different compared to control water (Glass *n* = 10, Plastic *n* = 10). **(B)** Fibrosis assessed comparing glass vs. plastic caging without BPA exposure in water bottles. Relative gene expression (RGE) of **(C,E)** collagen I (*Col1a1*) and **(D,F)** collagen III (*Col3a1*) vs. the housekeeping gene Hprt were analyzed in whole hearts by qRT-PCR at day 10 pi. **(C,D)** Compare 0–5 μg BPA/kg BW groups (0 BPA *n* = 9, 5 BPA *n* = 9). **(E,F)** Compare glass vs. plastic cages (glass *n* = 10, plastic *n* = 10).

Fibrosis is not present in the heart of male or female mice during acute CVB3 myocarditis at day 10 pi, and only begins to appear during chronic myocarditis around day 35 pi (54). We found that exposure to 5 or 50 μg BPA/kg BW in drinking water in mice housed in plastic cages significantly increased fibrosis in the heart of females with myocarditis (*p* = 0.007 and *p* = 0.01, respectively) using 2-tailed Student's *t*-test and 1-way ANOVA (all doses *p* = 0.04) (Figure 9A). BPA that may have leached from plastic cages and water bottles not only increased the number of mast cells in the heart (Figure 4A), but also significantly increased fibrosis in the heart compared to mice housed in glass cages (glass 9.8 ± 0.8 vs. plastic 13.8 ± 1.2, *p* = 0.01) (Figure 9B). We showed previously that mast cell degranulation is associated with increased fibrosis during myocarditis (50).

**Figure 10:**
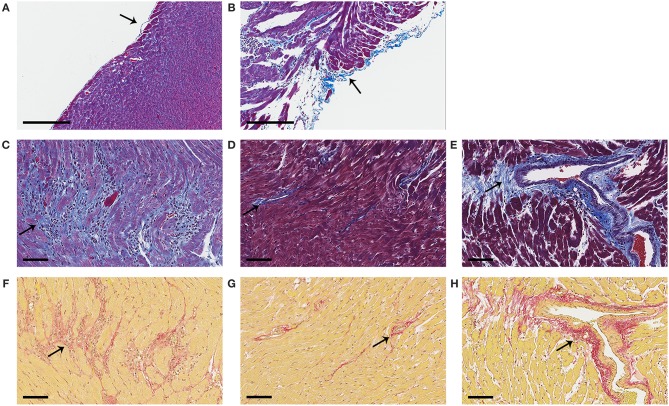
Representative photos of pericardial, myocardial and perivascular fibrosis. Female BALB/c mice were given 0, 0.5, 5, and 50 μg BPA/kg BW BPA in drinking water for 2 weeks and then injected ip with 10^3^ PFU of CVB3 ip on day 0 and exposure continued until harvest for myocarditis at day 10 pi. **(A–E)** Fibrosis was assessed using Masson's trichrome blue to detect collagen and fibrin deposition, which stains bright blue. **(F–H)** Picosirius red was used to assess collagen deposition, which stains red. **(A)** Normal pericardial layer; a single layer of pericardial cells lift from the myocardium and stain blue with Masson's trichrome. **(B)** Severe pericarditis with collagen (i.e., fibrosis) that stains bright blue. **(C)** Myocardial fibrosis staining bright blue or **(F)** red. **(D,G)** Normal myocardium with small amount of collagen staining mainly around vessels. **(E,H)** Perivascular fibrosis. Photos depict **(A,D,G)** 0 BPA, **(B,C,E,F,H)** 5 μg BPA/kg BW. **(A**, **B)** Magnification 100x, scale bar 200 μm. **(C–H)** Magnification 300x, scale bar 70 μm. Arrows point to staining for collagen.

Next we determined expression of collagen genes in the heart during myocarditis and found that BPA exposure in drinking water significantly increased expression of collagen I (*Col1a1*) (*p* = 0.005) (Figure 9C) and collagen III (*Col3a1*) (*p* = 0.02) (Figure 9D) compared to control water. We also assessed the effect of housing on collagen gene expression in the heart and found that plastic caging had no effect on the production of collagen I (*p* = 0.28) (Figure 9E) or collagen III (*p* = 0.67) (Figure 9F) during myocarditis. Mice exposed to BPA in drinking water also had greater pericardial (Figure 10B), myocardial (Figures 10C,F), and perivascular fibrosis (Figures 10E,H) compared to mice that had no BPA added to their water based on Masson's trichrome stain which uses three stains to identify muscle, collagen and fibrin (Figures 10A–E) and picosirius red which detects collagen [(55); Figures 10F–H].

## Discussion

In this study we found that BPA exposure at a high human relevant dose (25 μg BPA/L water or 5 μg BPA/kg BW) administered to adult female BALB/c mice significantly increased CVB3 myocarditis (Figure 1A) and pericarditis (Figure 2A) at day 10 pi compared to control water. The increase in myocardial and pericardial inflammation was not due to elevated levels of virus in the heart and, importantly, was not due to BPA that leached from the plastic cages or water bottles (Figures 1B, 2B). BPA exposure in water significantly increased ERβ expression in the heart (Figures 5B, 6C,D), which has been found to promote CVB3 myocarditis in male and female mice (10, 11), but was not significantly altered by plastic caging alone (Figure 5C). BPA also increased T cell numbers in the heart and proinflammatory and profibrotic cytokines (i.e., IFNγ, IL-17A, IL-1β) and signaling pathways (i.e., TLR4/IL-1R) that were not increased by BPA leaching from plastic cages and water bottles alone (Figures 7, 8). However, BPA (or other plastics/chemicals) leaching from plastic cages and water bottles was found to activate cardiac mast cells especially along the pericardium (Figures 3, 4) and to increase cardiac fibrosis (Figures 9, 10).

These findings have broad implications. First, the cages used for these experiments are the traditional cages used by many research facilities and investigator's experiments may be influenced by BPA leaching from cages and water bottles leading to mast cell activation. Our studies suggest that animal models that use viral infections to induce disease may be especially vulnerable to alterations caused by mast cell activation, although investigators studying the effects of allergy may also be affected by BPA leaching from caging materials. Second, women exposed to a high dose of BPA such as workers in factories that use plastic products as part of the manufacturing or packaging process, sales clerks handling receipts throughout the day, clerical staff that photocopy frequently, and/or certain furniture manufactures that use BPA resins in the production process, for example, may be at a higher risk of developing myocarditis following CVB infection. Individuals may not need a high lifetime exposure to BPA but an elevated exposure just prior to viral infection could be enough to increase their risk. The high use of plastics, especially associated with food and drink products, has made BPA and other plastic endocrine disruptors a ubiquitous exposure. Additionally, the discovery that BPA at higher doses in drinking water or leaching from plastic cages could increase fibrosis is important because female mice and women are highly protected from remodeling and fibrosis that leads to DCM and heart failure but we found in this study that remodeling and fibrosis are promoted by this endocrine disrupting chemical.

In our study, we administered BPA dissolved in drinking water based on Jenkins et al. (41) and found that a high human relevant exposure significantly increased myocarditis in our mouse model (Figure 1A). Animal studies researching the effect of BPA use many different exposure routes such as ip, gavage and by applying oil-based BPA to the chow of mice (56). We chose water as the exposure route as it has been found to be the most clinically relevant exposure route (25) and is a continuous exposure compared to using a high dose one-time injection/ bolus. People are exposed to BPA in many different ways including orally (i.e., drinking water from water bottles or eating food from aluminum cans coated with BPA) and by contact (by touching shiny receipts, fresh epoxy resin or through medical devices such as implants or tubing) (19–22).

In our BPA exposure experiments we housed all mice, both those exposed to BPA water and those receiving control water, in the same Allentown plastic standard mouse cages with plastic water bottles. The housing and water bottles are both made of plastics that contain BPA and other bisphenols that could leach from the caging (56–58). Studies have found that the age of cages can have a dose effect on the amount of BPA that is leached, with new cages releasing no more BPA than BPA-free or glass cages while older cages leach significantly more BPA (47, 56–58). The cages that we used for these experiments were not new and were all the same age. This means that the 0 BPA group is likely not truly zero because the mice were housed in older plastic cages. However, the potential BPA exposure from plastic cages was the same for all groups and our experiments comparing glass to plastic cages revealed the effect of the plastic cages and water bottles on our experimental endpoints.

At the time we started these studies we chose BPA doses based on Jenkins et al. who found that the 5 BPA dose was equivalent to a high human relevant exposure [(41); Table 1]. This dose of BPA is thought to be equal to exposures experienced by workers who are constantly exposed to BPA through their occupation as well as through personal exposures from food cans, water bottles, heating leftover food in plastic containers, etc. Occupations thought to have heavy exposure to BPA include cashiers (coated receipts), craftsman industries such as furniture design (epoxy resin), and canning companies (lining of cans), for example (19–22). Our data found that the highest dose of BPA (50 BPA), the EPA reference dose, did not significantly increase myocarditis (Figure 1A). Although most exogenous drugs/radiation/chemotherapy are thought to have linear dose-response curves, that is not the case for hormones (59, 60). The dose-response curve for hormones is a bell shaped curve that is termed hormesis because low and high concentrations of the hormone have a detrimental effect on health while a middle or moderate level is optimal for health (61). This principle may also apply to endocrine disrupting hormones such as BPA, where low and high levels of BPA have no detrimental effect on health but a middle or moderate exposure promotes disease (62).

Since BPA is known to act via ERs our data show that activating and/or altering the expression level of these receptors in/on immune or cardiac cells can increase the severity of autoimmune CVB3 myocarditis and promote pericarditis. Altering ER expression and/or ERα to ERβ ratios may also shift the type of immune response (i.e., Th2 to Th1/Th17A) following BPA exposure promoting a more cardio-damaging phenotype that increases inflammation, fibrosis and promotes progression to DCM. In this study we found that exposure to BPA in drinking water elevated proinflammatory cytokines/ receptors in the heart of female mice during CVB3 myocarditis that would typically be elevated in male mice or men with mycarditis such as TLR4, caspase-1, IL-1β, IL-17A, and IFNγ (44, 46, 63). Other investigators have reported that BPA exposure causes a shift from a Th2- (associated with IL-4) to a Th1- (associated with IFNγ) type immune response (64–66) which is similar to our findings. Thus, the immune response following BPA exposure resembles the cardiac inflammation characteristic of male mice with myocarditis suggesting that exposure to BPA could increase the risk of women developing myocarditis who may normally be protected by estrogen.

BPA exposure to myeloid immune cells from a mouse model of lupus or human peripheral blood cells was found to activate the TLR4 pathway causing the release of mature IL-1β and IL-18 (65). BPA treatment in cell culture of a monocyte-like cell line derived from a leukemia patient and human peripheral blood macrophages have been found to release the proinflammatory cytokines TNFα and IL-6 and to decrease the anti-inflammatory/ regulatory cytokines IL-10 and TGFβ (67). Lui et al. showed that this effect on cytokines by BPA was mediated through ERα and ERβ (67). Nowak et al. reviews many studies examining the effects of endocrine disrupting chemicals including BPA on immune cell function (68). We found that ERα and ERβ were activated during the innate immune response in the spleen and BPA has been found to increase IFNγ in mouse bone marrow cells and isolated CD11b^+^ cells via ERα (65), suggesting that BPA could be acting through ERs located directly on immune cells to shift the cytokine profile in the heart.

We found that BPA exposure significantly decreased ERα and increased ERβ expression in the heart of females during myocarditis (Figure 5B). A recent study in a rat model assessed the effect of BPA on the uterus and found that BPA treatment downregulated ERα and upregulated ERβ mRNA in a manner similar to our findings (16). Another study in mice found BPA increased ERβ levels which promoted cardiac arrhythmias and worse cardiac handling, while ERα was protective (13, 14). The opposing action of the two main ERs in these studies was investigated and it was found that ERα and ERβ transcriptionally cross-regulate each other (69, 70). But it is also thought that ERRγ, which BPA strongly binds to, can regulate ERα by heterodimerizing leading to transcriptional repression (71). Additionally, BPA can act as a ERβ antagonist rather than an agonist and prevent estrogen-driven non-genomic signaling pathways that could possibly be protective (70).

IL-17A, but not IFNγ, has been shown to promote remodeling and fibrosis and progression from acute myocarditis to chronic myocarditis and DCM in the CVB3 and experimental autoimmune models of myocarditis in male mice and in male patients with myocarditis (54, 63, 72). Activation of TLR4, caspase-1, IL-1β, and mast cells are also known to increase myocarditis and promote remodeling, fibrosis, and DCM in CVB3 myocarditis in male mice (44, 54) suggesting that BPA exposure could increase the risk of progression from myocarditis to DCM and heart failure in women. Importantly, ERα has been found to modulate TLR expression including TLR 2, 7, 8, and 9 (11, 27, 73, 74) while ERα has been found to decrease TLR4 (35).

Numerous studies have found that BPA and other endocrine disruptors activate mast cells leading to degranulation and release of histamine, leukotrienes and other mediators (68). Typically women have a reduced risk of progressing to DCM after myocarditis and are more likely to recover without the need for a heart transplant (4, 6). Other studies have found that BPA increases TLR expression in neonates (75) and promotes cardiac fibrosis (76, 77). Our findings in this study suggest that BPA exposure hastens the onset of remodeling and fibrosis, which usually takes several weeks to develop. In this study mice had not yet developed DCM even though they had pericardial and myocardial fibrosis.

We examined the effect of plastic cages/ water bottles to control for BPA that may leach from the plastic caging. We were surprised to find that exposure to plastic caging alone was able to increase the number and activation of mast cells and lead to pericardial degranulation and fibrosis. cKit is the receptor for stem cell factor which is a marker used to detect mast cells and stem cells (78). Previously, we found that mast cell degranulation was associated with increased CVB3 myocarditis, pericarditis, DCM, and heart failure in male BALB/c mice (54, 79). Mast cells leave the bone marrow as undifferentiated cells and migrate to the heart where they differentiate to form two types of resident mast cell populations: one type contains tryptase and chymotrypsin (also called Serpin A3n) granules and is termed a *TC mast cell* and the other type of mast cell contains only tryptase granules and is termed a *T mast cell* (80). It is not known whether one type of mast cell resides along the pericardium and the other in the myocardium, but this is a possibility. To determine whether BPA exposure altered degranulation of mast cells located in the myocardium we examined the degranulation state of myocardial mast cells using histology. Mast cells that occur in the circulation are known as basophils. Basophils can be recruited to sites of inflammation (81). Infiltrating mast cells or basophils would be expected to be found near vessels. Mast cells found near vessels have been termed “vessel-associated” mast cells for this analysis and are assumed to be mast cells recruited to the heart from the circulation rather than resident mast cells. Mast cells in the myocardium that were not near vessels or evidence of red blood cells were defined as “myocardial” mast cells. It is likely that there is a certain amount of overlap between “myocardial” and “vessel-associated” mast cells due to the inability to detect vessels depending on the cut of the histology section. To determine whether BPA exposure from drinking water or plastic cages altered degranulation of mast cells located near vessels we examined the degranulation state of “vessel-associated” mast cells using histology. Previously, we found that pericardial mast cell degranulation was associated with increased pericarditis, immune complex deposition along the pericardium, DCM and, heart failure in mice (54, 79). BPA exposure at concentrations relevant to human exposure, like in this study, have been found to enhance histamine and leukotriene release from bone marrow-derived mast cells but not did not require ER signaling for this effect.

Overall, our findings suggest that BPA exposure at higher doses increases the risk for women to develop myocarditis following CVB3 infection. Exposure to BPA that has leached from plastic containers activates mast cells in the heart in the context of viral infection, particularly along the pericardium. Future studies will be needed to determine whether the increase in myocarditis caused by BPA is due primarily to ER activation/alteration on immune or cardiac cells.

## Data Availability

The raw data supporting the conclusions of this manuscript will be made available by the authors, without undue reservation, to any qualified researcher.

## Ethics Statement

Mice were used in strict accordance with the recommendations in the Guide for the Care and Use the Laboratory Animals of the National Institutes of Health. Mice were maintained under pathogen-free conditions in the animal facility at the Johns Hopkins School of Medicine and at Mayo Clinic Florida, and approval obtained from the Animal Care and Use Committee at Johns Hopkins University and Mayo Clinic Florida for all procedures. Mice were sacrificed according to the Guide for the Care and Use of Laboratory Animals of the National Institutes of Health.

## Author Contributions

KB and DF designed the study, generated the figures, and wrote the paper. KB, JM, AY, JF, AJS, HG, FM, MG, GC, AB, AM-L, AH, AM, DD, AC, and ARS performed the experiments. KB, JS, and DF analyzed the data. KB, AM-L, AH, AM, DD, JS, and DF edited the paper.

### Conflict of Interest Statement

The authors declare that the research was conducted in the absence of any commercial or financial relationships that could be construed as a potential conflict of interest.
